# Patterns of Cannabis use and co-occurring substance use in migrants with psychiatric disorders

**DOI:** 10.1192/j.eurpsy.2026.10820

**Published:** 2026-07-31

**Authors:** F. Sahnoun, S. Omri, N. Smaoui, N. Charfi, I. Gassara, R. Feki, M. Maalej, M. Maalej Bouali, L. Zouari

**Affiliations:** Psychiatry C Department, Hedi Chaker University Hospital, Sfax, Tunisia

## Abstract

**Introduction:**

Cannabis use among migrants with psychiatric disorders is often accompanied by other substance consumption. Understanding these associations and their interrelations is essential for designing effective prevention and intervention strategies.

**Objectives:**

To explore the characteristics of cannabis use and associations with other substance use in a population of migrants hospitalized in psychiatry.

**Methods:**

A cross-sectional descriptive and analytical study was conducted among patients hospitalized in the Psychiatry “C” Department of Hédi Chaker University Hospital in Sfax. Participants had previously lived abroad and experienced the onset of psychiatric disorders during their stay. Data were collected via a structured questionnaire including sociodemographic characteristics, migration history, cannabis use, and use of other substances, as well as involvement in drug-related activities.

**Results:**

A total of 39 male immigrants participated in the study. The mean age was 35.9 years. The most frequently identified diagnosis was schizophrenia (35.9%), followed by bipolar disorder (28.2% of cases). Among the participants, 69.2% reported using cannabis during their stay abroad. The mean age of first cannabis use was 19.2 years, and for 55.6% of users, consumption began before migration. All cannabis users reported using the substance before the onset of psychiatric disorders.

Nearly half of the users (48.1%) reported daily consumption. The most commonly reported effect sought was anxiety reduction (51.9%). Cannabis was consumed in the form of joints in all cases, and 55.6% of participants used it alone. In addition to cannabis, participants reported using other substances such as cocaine at 28,2%, ecstasy at 12.8%, pregabalin at 10.3 %, and heroin at 7.7%.

Additionally, 35.9% reported involvement in drug trafficking, motivated essentially by financial difficulties.

Cannabis use was significantly associated with tobacco use (p = 0.024), cocaine consumption (p = 0.008), and other psychoactive substances consumption (p = 0.001).

**Conclusions:**

Cannabis use among migrants with psychiatric disorders is closely linked to other substance use. These findings underline the need for comprehensive addiction assessment and integrated intervention strategies that address multiple substances and behavioral risks in this vulnerable population.

**Disclosure of Interest:**

None Declared

